# Content Validation and Semantic Evaluation of a Check-List Elaborated for the Prevention of Gluten Cross-Contamination in Food Services

**DOI:** 10.3390/nu9010036

**Published:** 2017-01-06

**Authors:** Priscila Farage, Renata Puppin Zandonadi, Verônica Cortez Ginani, Lenora Gandolfi, Riccardo Pratesi, Yanna Karla de Medeiros Nóbrega

**Affiliations:** 1 Department of Nutrition, Faculty of Health Sciences, University of Brasilia (UnB), Campus Darcy Ribeiro, Asa Norte, Brasilia DF 70910-900, Brazil; renatapz@yahoo.com.br (R.P.Z.); vcginani@gmail.com (V.C.G.); 2 Faculty of Medicine, University of Brasilia (UnB), Campus Darcy Ribeiro, Asa Norte, Brasilia DF 70910-900, Brazil; lenoragandolfi1@gmail.com (L.G.); pratesiunb@gmail.com (R.P.); yannanobrega@gmail.com (Y.K.d.M.N.)

**Keywords:** gluten, gluten contamination, food safety, celiac disease, gluten related disorders

## Abstract

Conditions associated to the consumption of gluten have emerged as a major health care concern and the treatment consists on a lifelong gluten-free diet. Providing safe food for these individuals includes adapting to safety procedures within the food chain and preventing gluten cross-contamination in gluten-free food. However, a gluten cross-contamination prevention protocol or check-list has not yet been validated. Therefore, the aim of this study was to perform the content validation and semantic evaluation of a check-list elaborated for the prevention of gluten cross-contamination in food services. The preliminary version of the check-list was elaborated based on the Brazilian resolution for food safety *Collegiate Board Resolution 216* (RDC 216) and *Collegiate Board Resolution 275* (RDC 275), the *standard 22000* from the International Organization for Standardization (ISO 22000) and the Canadian Celiac Association *Gluten-Free Certification Program* documents. Seven experts with experience in the area participated in the check-list validation and semantic evaluation. The criteria used for the approval of the items, as to their importance for the prevention of gluten cross-contamination and clarity of the wording, was the achievement of a minimal of 80% of agreement between the experts (W-values ≥ 0.8). Moreover, items should have a mean ≥4 in the evaluation of importance (Likert scale from 1 to 5) and clarity (Likert scale from 0 to 5) in order to be maintained in the instrument. The final version of the check-list was composed of 84 items, divided into 12 sections. After being redesigned and re-evaluated, the items were considered important and comprehensive by the experts (both with W-values ≥ 0.89). The check-list developed was validated with respect to content and approved in the semantic evaluation.

## 1. Introduction

Recently, there has been a growing demand for gluten-free products in the world population. The global market of these products approached $2.5 billion (US) in sales in 2010. It seems that the number of individuals embracing a gluten-free diet (GFD) is much higher than the projected number of celiac disease (CD) patients. This finding can be explained by the existence of Gluten Related Disorders (GRD) other than CD, which is now clear. The GRD include three main forms of gluten reactions: allergic (wheat allergy), autoimmune (CD, dermatitis herpetiformis, and gluten ataxia), and possibly immune-mediated (gluten sensitivity) [1].

Despite differences in pathological mechanisms, clinical manifestations, and epidemiology, the treatment for all GRD consists of excluding gluten-containing cereals and sub-products from the diet. Combined, these conditions affect many individuals who consequently need to follow the GFD. CD accounts for around 1% of the general population. In regards to wheat allergy, different prevalence rates have been found in studies around the world, varying from 0.4% in adults to as high as 9% in children [1]. The prevalence of gluten sensitivity is not clearly defined yet. However, indirect evidence suggests that it is slightly more common than CD [2].

According to Codex Alimentarius, “gluten-free foods” (GFF) are those in which the gluten level does not exceed 20 ppm (mg/kg) in total [3]. In a systematic review, Akobeng et al. (2008) [4] investigated the threshold amount of gluten that could be tolerated by people with CD and found that there is a variation among individuals. Although there was no evidence to suggest a single definitive threshold, they found that a daily gluten intake of less than 10 mg was unlikely to cause significant histological abnormalities in celiac patients [4]. As to the other GRD, further studies are necessary to clarify whether the spectrum of toxic cereals, the gluten threshold, and the disease duration are the same as in CD, since their natural history, particularly of gluten sensitivity, is still unclear [1].

Following the GFD is a difficult task for GRD patients due to the presence of gluten in a wide range of products. Moreover, gluten may be found in supposedly gluten-free products as a consequence of cross-contamination, which leads to the involuntary and unconscious consumption of it [5].

Cross-contamination might occur because of shared production areas, kitchenware not properly sanitized, and inadequate procedures by restaurant staff [6]. In most countries there is not a consistent monitoring process to assess gluten content in supposedly GFF in order to guarantee safe products for CD and other GRD patients [7]. Moreover, studies have revealed gluten-contamination in both industrial products and food services preparations [8,9,10], which represents a problem for these patients since maintaining gluten in the diet triggers symptoms and health problems such as gastrointestinal manifestations and other related conditions [1].

Therefore, eating out may be considered a health risk for GRD individuals [11] and the need to follow the GFD may compromise social activities and influence quality of life [12]. Thus, in order to contribute to a better quality of life for GRD patients, it is important to establish viable and effective strategies to prevent contamination and enable the safe production of gluten-free food [11].

The development of an instrument for the verification of non-conformities in loco that are related to the occurrence of cross-contamination seems like an interesting approach in order to control the production process and provide safe food for GRD patients considering the paucity of studies that investigate possible strategies to prevent gluten cross-contamination in food services.

In a study conducted in Italy, the Hazard Analysis and Critical Control Point system (HACCP) was used for the elaboration of a plan to prevent gluten contamination in a school cafeteria, and the results showed the effectiveness of this plan in the reduction of contamination [13]. In Brazil, Bicudo (2010) [14] elaborated and implemented the standard operating procedures (SOPs) for items related to accidental gluten cross-contamination in a bakery. In this study, a checklist was first applied followed by the elaboration of corrective measures for problems found. The association of the SOPs together with the corrective measures based on good manufacturing practices was effective in controlling gluten contamination in the study site [14].

Most studies on food safety discuss issues related to microbiological contamination, however, it is important that food services adapt to food preparation practices in order to produce safe special diets, such as the GFD.

There are specific regulations on gluten-free labelling in the context of gluten intolerance worldwide. Most of them are based on the Codex Alimentarius Standard 118-1979 and recommend following good manufacturing practices for the prevention of gluten cross-contamination, ranging from country to country. The European Union, United States, and Canada follow the limits proposed by Codex for GFF (20 ppm). In Argentina, the threshold set for GFF is 10 ppm. In Australia and New Zealand, legislation is stricter and states that to be considered “gluten-free”, food must not contain detectable gluten [7,15,16]. However, it must be emphasized that, in food services, this is rarely regulated and monitored.

For the development of an instrument for data collection, the phenomena of interest must be translated into concepts that can be measured, observed, or recorded. Without proper methods for data collection, the validity of the questionnaire conclusions is questionable. Thus, it is very important to consider some points during the process, such as, an extensive review of literature on the theme, experience of the researcher on the subject, care and monitoring of the formulation of each question/item regarding clarity, consistency, relevance, and impartiality; evaluation of the instrument by experts in the field of knowledge, and the testing to verify whether the instrument is useful in order to obtain the desired information [17].

The validation of an instrument consists of a methodological procedure to evaluate its quality, which is related to the capacity of the instrument to accurately measure what it is intended to measure [17]. The content validity refers to the representativeness and relevance of the instrument questions. The content validation can be analyzed by a panel composed of professionals and researchers recognized in their area [18]. The expert panel consensus helps defining the instrument items which should be maintained, revised, or excluded and its application is increasing in several areas [19].

Another important procedure to obtain a satisfactory instrument is to perform the semantic evaluation, which measures the comprehension of the instrument items by the judges and helps to evaluate the need to rewrite the questions in order to achieve a better understanding of the instrument [20].

This study aimed to perform the content validation and semantic evaluation of an instrument (check-list) elaborated for the prevention and control of gluten cross-contamination in food services.

## 2. Methods

### 2.1. Development of the Instrument

The instrument (check-list) was elaborated based on extensive literature review and experience of the researchers on the matter. The following documents were used to design the preliminary version of the check-list: the Brazilian resolutions for food safety *Collegiate Board Resolution 216* (RDC 216) and *Collegiate Board Resolution 275* (RDC 275), the international *standard 22000* from the International Organization for Standardization (ISO 22000), and the documents from the *Gluten-Free Certification Program*, of the Canadian Celiac Association [21,22,23,24].

Topics and items from the resolutions RDC 216 and RDC 275 and the ISO 22000 standard were carefully evaluated and those thought to be relevant to the prevention of gluten cross-contamination were chosen and adapted for the initial version of the check-list, even though these documents do not specifically address the prevention of gluten cross-contamination. However, the premise of a functional gluten contamination control system is based on prerequisites programs implemented in the establishment, attending minimally to the good manufacturing practices, as proposed by the Codex [3].

Important topics of the *Gluten-Free Certification Program,* Canada, were also selected to compose the check-list and adapted to contemplate the reality of food services. The preliminary version was composed of 136 items divided into 13 major sections, listed below:

- Identification/information of the establishment
- Building and facilities
- Equipment, furniture, and kitchenware
- Food service employees
- Food production and transport
- Distribution
- Documentation
- Responsibility and authority
- Coordinator of the food safety team
- Internal communication
- Flow charts
- Traceability
- Treatment of potentially unsafe products

All of the items had a “Yes/No/Not Applicable” type of answer, such as the check-list presented in the RDC 275, except for the items of the “Identification/information of the establishment” section, which contains open questions to characterize the establishment (name of the place, address, owner, among others).

### 2.2. Pilot Test (Subjective Evaluation)

For the content validation, a total of 11 experts with a PhD and known experience in instruments of quality control for food services and/or gluten and CD were invited to participate. A total of seven experts were available for the study. The experts received the necessary information and guidance on the check-list method of evaluation. The check-list was sent by e-mail.

At first, experts were asked to express their opinion on the preliminary version of the instrument and evaluate the overall questionnaire, considering aspects such as the content, clarity, type, and consistency of the items. Experts were also asked to suggest any modification, exclusion, or inclusion of items they judged relevant and to freely comment on any subject regarding the instrument. This was characterized as a qualitative analysis stage.

### 2.3. Content Validation

The Delphi method was used, with some adaptations, for the content validation. This method is based on obtaining the opinions of experts in order to achieve a consensus on a specific subject. The Delphi method is currently employed in several areas in situations where new ideas are being created. It is a method in which, through collegial communication ordered by individual responses, often conducted by questionnaires, we seek the consensus of a group [19].

The Monkey Survey^@^ platform was used to create a questionnaire for the application of the content validation of the check-list. On the first page of the questionnaire there was an orientation letter specifying the evaluation criteria for the check-list items. Experts were asked to evaluate each item considering its importance for the prevention of gluten cross-contamination using a Likert scale, as follows: (1) “I totally disagree with the item”; (2) “I partially disagree with the item”; (3) “I neither agree nor disagree with the item”; (4) “I partially disagree with the item”; and (5) “I fully agree with the item”.

The Monkey Survey^@^ platform was also used to provide feedback to the experts in regards to the evaluations performed by other experts and final results of the analysis. Two stages of evaluation were performed in the content validation process. For the items which did not receive approval in the first stage, the means resulting from the experts’ opinions were presented to each one of them. After being informed about the other experts’ opinions, the experts were asked to review their analysis and decide whether or not they would confirm previous answers. This procedure was performed in order to obtain consensus among the experts. All seven experts participated in this phase.

### 2.4. Semantic Evaluation

The semantic evaluation of the check-list was performed simultaneously with the content validation, using the same questionnaire in the Monkey Survey^@^ platform. Experts were asked to evaluate each item in regards to its clarity, considering their level of understanding of the item. For that purpose, the Likert scale was used, as follows: (0) “I did not understand it at all”; (1) “I understood it a little”; (2) “I somewhat understood it”; (3) “I understood almost everything, but I had some questions”; (4) “I understood almost everything”; (5) “I understood it perfectly and had no questions”. According to Conti et al. (2010) [20], answers from 0 to 3 indicate insufficient understanding and a new version of the item is required [20].

In cases of poor understanding of the item or unsuitable language, experts were also asked to suggest changes. These commentaries were used to create new versions of the items for further evaluation. Three stages of evaluation were performed in the semantic evaluation process. Six experts participated in the last stage.

### 2.5. Data Analysis

For data analysis, all answers obtained with the questionnaire were compiled using the Microsoft Excel 97-2003 software.

The mean grade for the evaluation of importance and clarity of each item was calculated considering the answers provided by the seven experts, except for the last stage of the semantic evaluation, in which six experts participated. The degree of agreement among the experts for the evaluation of importance and clarity of the items was evaluated through the Kendall (W) coefficient of concordance, which ranges from 0 to 1. High W-values (W ≥ 0.66) indicate that the experts applied the same standards of evaluation as opposed to Low W-values, which suggest disagreement among the experts [17].

The criteria established for the approval of the item was a minimal of 80% of agreement between the experts (W-values ≥ 0.8). Moreover, items should have a mean ≥4 for the evaluation of importance (content validation) and clarity (semantic evaluation) in order to be maintained in the instrument. Items not considered important for the prevention of gluten-cross contamination in food services were excluded from the instrument. Items considered unclear were rewritten in a different manner and subject to further evaluation by the experts.

Suggestions made by the experts were considered and incorporated into the final version of the instrument.

## 3. Results

Considering the suggestions made by the experts in the pilot test, a new version of the check-list was created, consisting of 88 items, divided into 12 sections. The “Traceability” section was not considered applicable for the food service environment and it was removed from the check-list. This new version was then submitted to an objective evaluation. At this point, the first stage of the content validation and the semantic evaluation was performed. In total, two stages of evaluation were necessary in order to obtain agreement among the experts for the content validation and three stages were necessary for the semantic evaluation.

**Figure 1:**
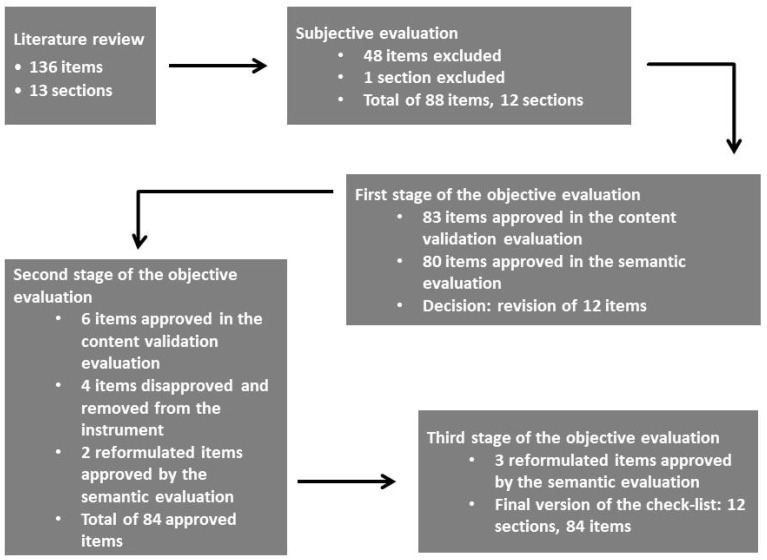
Stages of the content validation and semantic evaluation processes.

The summary of stages and exclusion or corrections of items of the whole validation process are displayed in Figure 1.

### 3.1. First Stage: Content Validation and Semantic Evaluation

In the first evaluation of the content validation process, a total of 83 items (94.3%) were approved, that is, there was a minimal of 80% of agreement between the experts (W-values ≥ 0.8) and the items displayed a mean ≥4 in the evaluation of importance. The remaining five items without approval in this stage were: 1.6.2 (regarding goods lift for gluten-free food), 1.8.1 (regarding washbasins and soap supply in the production area), 1.12.1 (regarding the proper layout for food production), 8.5 (regarding the report on effectiveness and adequacy of the control of gluten contamination by the coordinator of the food safety team), and 9.8 (regarding information about relevant issues from outside concerned parties).

As to the semantic evaluation, a total of 80 items (90.9%) were considered sufficiently understandable (these items received grades “4” or “5” in the Likert scale) and thus were approved without needing to adjust the wording.

The mean grades and W-values for each section, considering the means of all items, for the content validation and semantic evaluation are presented in Table 1.

Despite being approved in regards to the content validation in stage 1, items 9.7 (regarding information about customer requirements, sectoral requirements, and others), 9.9 (regarding information about customer complaints indicating food safety hazards associated with the product), 9.10 (regarding information about other conditions which might impact the gluten contamination control), 9.11 (regarding update of the gluten contamination control system), and 9.12 (regarding the inclusion of relevant information for critical analysis in the system) were not considered clear enough by the experts in the semantic evaluation.

Moreover, some experts suggested the removal of some of those items and made comments about the lack of understanding of the purpose of the item in the check-list and how to verify what it proposed regarding food service practices. They also mentioned that some of those items were too subjective and/or repetitive. Therefore, researchers considered it important to resubmit these items to the evaluation of importance in the instrument, through a new evaluation of content, before rewriting the items and submitting them to a new semantic evaluation.

Thus, a total of 12 items were subject to further evaluation in stage 2.

### 3.2. Second Stage: Content Validation and Semantic Evaluation

In this stage, items 1.6.2, 1.8.1, 1.12.1, 8.5, 9.7, 9.8, 9.9, 9.10, 9.11, and 9.12 were submitted once more to the content validation. For that purpose, the means of grades attributed by the experts in the previous stage were presented to them in order for them to check whether they wanted to maintain the grade that was previously assigned to the item or whether they wanted to reconsider taking into consideration the opinion of the other experts. A sum of comments made by the experts was also presented for them to help achieve a consensus.

At this point, six of these items (60%)—1.6.2, 1.8.1, 1.12.1, 9.9, 9.10, and 9.11—were considered important by the experts and thus maintained in the check-list. The other four items—8.5, 9.7, 9.8, and 9.12—were removed from the check-list (mean grade < 4).

Items 1.6.1 (regarding ramps and workbenches) and 1.11.1 (regarding containers for the collection of waste within the facility) were not considered sufficiently understandable in stage 1. These items were reformulated considering comments and suggestions made by the experts in stage 1 and subject to semantic evaluation. Both of them were approved in this new version.

Since items 9.9, 9.10, and 9.11 were reassessed by the experts as to their importance for the prevention of gluten cross-contamination and received grades >4, they were kept in the check-list. However, they had not been approved as to their clarity in the first stage of the semantic evaluation. Therefore, these items were subject to a new stage of semantic evaluation.

### 3.3. Third Stage: Semantic Evaluation

At this point, only three items—9.9, 9.10, and 9.11—needed further evaluation, in regards to their clarity. The items were reformulated based on previous comments and suggestions by the experts. In this stage, one expert was not available to participate and the mean grades were calculated based on the other six experts’ opinions. The new versions of the items were approved in this stage and the process of content validation and semantic evaluation was accomplished. It is important to mention that the content validation and semantic evaluation were performed in Portuguese, the original version of the instrument. However, the complete check-list (Appendix A) was translated into English in order to facilitate the readers’ understanding. It can be found in the appendix section.

## 4. Discussion

Gluten contamination in supposedly gluten-free food is a very concerning issue. As the study by Hollon et al. (2013) [25] showed, gluten traces may impair histological and clinical recovery of patients, even leading to an incorrect diagnosis of refractory celiac disease (RCD), which would result in the unnecessary use of corticosteroids or immunotherapy with potential adverse health effects [25].

The most common cause of non-response in the treatment for CD is related to the failure to adhere to the GFD [25], including unintentional consumption of gluten by means of contaminated food. This fact highlights the importance of providing safe food for CD patients.

In the process of development and validation of an instrument, it is very important to use rigorous methods [17]. In this study, the Delphi technique was chosen. It allows the implementation of an experts panel in order to perform the content validation, facilitating the achievement of consensus on the experts’ opinions [26].

As in the study by Ceniccola et al. (2014) [26], the Delphi technique was used to guide the stages of the experts’ evaluations, making them interact with the research group through structured rounds. As mentioned earlier, this was performed using the Survey Monkey^@^ platform, which enables the provision of feedback to the experts. The feedback is proposed in the Delphi technique as it helps to assure a more organized interaction with the experts [26].

The appropriate selection of the experts is also a critical point to obtain solid results and it is based on the experience and the knowledge of the participants in a certain area, besides the willingness to collaborate with the study. There is no consensus in the literature in regards to the number of experts to perform the validation process [19,27,28,29,30]. Nevertheless, Pasquali (1999) [28] considers that a minimum of six experts is necessary to reach a consensus, although this number may vary according to the type of the instrument [28]. In this study, a total of seven experts participated.

The obtaining of a validated check-list for the control of gluten contamination is of urgent need for food services. In Brazil, hygienic-sanitary control in food production has been improving in recent years. The rules defined in resolutions on the subject have proved to be effective, since a lot of studies have shown the reduction of outbreaks of foodborne diseases [31]. However, there is a lack of studies on the development of quality control instruments for the prevention of gluten cross-contamination. Despite the fact that the Brazilian legislation sets the obligatoriness when including a statement regarding the presence or absence of gluten in the label of industrial products, it does not address the production of gluten-free food in food services [8].

In this study, a check-list was elaborated and evaluated with the purpose of providing an appropriate tool to assist in the gluten-free food production system and ensuring the right to safe food for GRD patients. The final check-list was carefully revised and all items included were considered important and comprehensive by the experts (both with agreement by Kendall coefficient ≥0.89).

The check-list created presents strong points, since it was submitted to the evaluation of experts on the area, who were free to make any comments which they deemed relevant to improve the instrument. Moreover, the semantic evaluation process helps to ensure that the items are clear and comprehensive as to the language and writing.

As a study by Araújo et al. (2011) [32] revealed, individuals who follow a GFD ingest food with gluten because of lack of alternatives and/or information in food found in public places [32]. Having a meal in a restaurant creates a problem for those individuals because of the lack of knowledge by the restaurant staff concerning the correct procedures to prevent contamination and provide safe food [33]. In a study conducted in Brazil, Laporte et al. (2011) [34] interviewed restaurant chefs regarding their knowledge about CD and only 30% of the participants referred knowing the disease [34].

Machado et al. (2013) assessed adherence to the GFD by structural interviews with CD patients and the results were compared to their IgA anti-transglutaminase antibodies’ levels. The serological tests showed that 56.5% of the individuals did not follow the GFD. However, 60.9% referred complete elimination of gluten from the diet. Among those, 35.7% presented a positive result in the serological test, which possibly indicates involuntary diet transgression [35].

This fact compromises social activities which ultimately impair quality of life [5]. Thus, viable and effective strategies to prevent contamination must be developed, including quality control audits to assure that established protocols are being followed. This has already been accomplished for the control of microbiological contamination and there is an urgent need to enable the same for the control of gluten cross-contamination.

Although there are other available check-lists for the control of gluten contamination, this study brings a novelty that is the validation of a specific tool for food services. Moreover, the use of the Delphi method allows for the ability to have a great volume of information; better reflection on the subject and more elaborated answers due to the use of questionnaires; elimination of influences of judgment that could interfere with the quality of the answers due to the anonymity of the technique; and the possibility of incorporating new ideas raised by experts in the area [19]. The semantic evaluation performed also makes this check-list an interesting tool since it helps to assure proper understanding of the items, which is crucial for the correct evaluation of conformities/non-conformities situations in loco and ultimately might impact the safety of the food produced in certain establishments.

This study is part of a larger study currently in progress. The check-list will be applied in food services where samples will be collected for the evaluation of gluten contamination. Data obtained will be submitted to statistical analysis to determine which items/sections are in fact related to the contamination and which trigger higher chances of generating contaminated food. Thus, in this second phase, it will be possible to evaluate the removal of unnecessary items from the check-list—which will make the check-list shorter and more practical—and also provide different grades to each item/section which will culminate in a score for classifying the establishment as to its risk of providing contaminated food.

The proposed check-list is attractive for its practicality and low cost. Moreover, it can be used for identifying inappropriate routines and allowing the correction of non-conformities to ensure safe food for those who need to engage a GFD.

## 5. Conclusions

The instrument (check-list) developed for the verification of non-conformities related to gluten-contamination in food services was validated with respect to content, after careful revision of its items. After it was redesigned, the items were considered important and comprehensive by the experts (both with agreement by Kendall coefficient ≥0.89).

However, it is important to highlight that future studies are necessary to assess other properties of the instrument, such as reliability using the criteria of reproducibility which aims at verifying the proportion of agreement among the responses when the instrument is applied in the same location and circumstances by different professionals.

Further studies are also necessary in order to test this instrument in food services and evaluate its effectiveness in contributing to the prevention of gluten cross-contamination. Strategies such as this are very important to improve the access to safe food by GRD patients and ultimately contribute to greater quality of life.

## Figures and Tables

**Table 1:** Experts evaluation of the check-list—mean grades and Kendall coefficient of concordance of the check-list sections.

Section of the Check-List	Content Validation (Mean Grade ± SD *)	Content Validation (W-Value)	Semantic Evaluation (Mean Grade ± SD *)	Semantic Evaluation (W-Value)
Building and facilities	4.74 ± 0.30	0.96	4.76 ± 0.15	0.92
Equipment, furniture and kitchenware	4.79 ± 0.25	0.97	4.83 ± 0.17	0.96
Food service employees	4.81 ± 0.20	0.98	4.79 ± 0.20	0.93
Food production and transport	4.79 ± 0.21	0.96	4.87 ± 0.17	0.98
Distribution	4.86 ± 0.14	0.94	5.00 ± 0.00	1.00
Documentation	4.82 ± 0.27	0.96	4.75 ± 0.32	0.96
Responsibility and authority	4.86 ± 0.00	1.00	5.00 ± 0.00	1.00
Coordinator of the food security team	4.57 ± 0.26	0.89	5.00 ± 0.00	1.00
Internal communication	4.78 ± 0.23	0.92	4.71 ± 0.29	0.94
Flow charts	4.86 ± 0.14	1.00	4.71 ± 0.14	0.90
Treatment of potentially unsafe products	4.52 ± 0.24	0.90	4.76 ± 0.20	0.95

* Standard Deviation.

## Appendix A

Check-List for the Verification of Non-Conformities Related to Gluten-Contamination in Food Services

**Legend:**			
**Y—Yes**	**N—No**	**NA—Not Applicable**	**OBS—Observation**

**Number:**				**Year:**				
**Company identification:**								
**Company name:**								
**Trading name:**								
**Health license:**				**State/municipal registration:**				
**National record of legalized person/individual registration:**				**Phone:**	**Fax:**			
**E-mail:**								
**Address:**								
**Neighborhood:**				**City:**	**State:**		**Zip code:**	
**Activity branch:**					**Monthly output:**			
**Number of employees:**					**Number of shifts:**			
**Products’ categories:**								
**Category description:**								
**Technical manager:**				**Academic background of the technical manager:**				
**Is there an employee responsible for the good manufacturing practices in the establishment?** **( ) Yes ( ) No**				**Academic background of the employee responsible for the good manufacturing practices:** **( ) training course** **( ) technical course. Which?** **( ) college degree. On what?**				
**Legal representative/owner of the establishment:**								
**Items**					**Y**	**N**	**NA**	**OBS**
**1.**	**Building and Facilities**							
	**1.1.**	**floor**						
		1.1.1.	Floor material that allows easy and proper sanitation (smooth, drained with slope, waterproof).					
		1.1.2.	Floor in proper conservation (free of defects, cracks, holes, and others).					
	**1.2.**	**Ceiling**						
		1.2.1.	Ceiling easy to clean waterproof with smooth finishing.					
	**1.3.**	**Walls**						
		1.3.1.	Smooth finishing walls, impermeable and easy to clean at suitable height for all operations.					
		1.3.2.	Wall in proper conservation (free from cracks and peeling).					
	**1.4.**	**Doors**						
		1.4.1.	Smooth surface doors, adjusted to the jambs and without coating faults in order to reduce the risk of contamination coming from the external area.					
	**1.5.**	**Windows and other openings**						
		1.5.1.	Smooth surface windows, adjusted to the jambs and without coating faults in order to reduce the risk of contamination coming from the external area.					
	**1.6.**	**Stairs, service elevators, goods lift, and auxiliary structures**						
		1.6.1.	In case of ramps and workbenches used to support both gluten-free and gluten-containing food, a hygienic procedure is performed between the use of this surface for gluten-containing and gluten-free food.					
		1.6.2.	There is a goods lift exclusive for the use of gluten-free food.					
	**1.7.**	**Toilets and dressing rooms for employees**						
		1.7.1.	Toilets equipped with washbasins and products intended for personal hygiene: antiseptic odorless liquid soap or odorless liquid soap and antiseptic, non-recycled paper towel or other safe and hygienic drying system, collectors with lid and without manual activation.					
	**1.8.**	**Washbasins in the production area**						
		1.8.1.	Existence of washbasins in the production area with running water, in appropriate positions in relation to the production and service flow, with sufficient number to suit the entire production area, preferably equipped with automatic stopcock, antiseptic odorless liquid soap or odorless liquid soap and antiseptic, non-recycled paper towels or other hygienic and safe drying system and paper collectors without manual activation.					
	**1.9.**	**Ventilation and air conditioning**						
		1.9.1.	Artificially air-conditioned environments, without fans, without generating airflow and absence of natural airflow from the production area of gluten-containing food to the production area of gluten-free food, avoiding an environment with particles in suspension.					
	**1.10.**	**Cleaning of the facilities**						
		1.10.1.	Facilities kept under appropriate hygienic-sanitary conditions, that is, without the presence of accumulation of residues, with proof by means of registration in specific spreadsheets, updated and with information consistent with what is being observed.					
		1.10.2.	Utensils used for the cleaning of facilities distinct from those used for the cleaning of equipment that come into contact with food, with hygiene products and utensils exclusive for the use in the production area of gluten-free food.					
	**1.11.**	**Waste management**						
		1.11.1.	Containers for the collection of waste inside the establishment which are easily sanitized (i.e., without cracks that allow dirt to accumulate and are difficult to access by cleaning utensils) and transported (i.e., can be easily moved by those responsible for the procedure); emptied whenever its content reaches 2/3 of its capacity and constantly sanitized, showing no evidence of accumulated dirt; use of appropriate garbage bags.					
		1.11.2.	Waste removed from the gluten-containing food production area does not pass through the production area of gluten-free food.					
	**1.12.**	**Layout**						
		1.12.1.	Layout suitable for the productive process: number, capacity, and distribution of dependencies according to the branch of activity, production volume, and expedition.					
		1.12.2.	Areas for receiving and depositing ingredients distinct from the areas of production, storage, and expedition of the final product.					
		1.12.3.	Gluten-free ingredients warehouse identified and in a different space from that of gluten-containing ingredients.					
		1.12.4.	Area of production of gluten-free food identified and in a separate space from that of the production area of gluten-containing food.					
**2.**	**Equipment, furniture, and kitchenware**							
	**2.1.**	**Equipment**						
		2.1.1.	Equipment arranged in a way that allows easy access and proper cleaning.					
		2.1.2.	Equipment with contact surfaces which are smooth, undamaged, waterproof, and easy to clean.					
		2.1.3.	Production line equipment (mixers, processors, blenders, toasters, etc.) identified and exclusive to the production of gluten-free food.					
		2.1.4.	Food preservation equipment (refrigerators, freezers, cold rooms) exclusive for gluten-free products or, when not possible, the disposal of products is done in separate spots and/or with some kind of physical separation between gluten-free and gluten-containing products.					
		2.1.5.	Thermal processing equipment (ovens) exclusive for gluten-free food or, when of common use, not used for baking gluten-free and gluten-containing food simultaneously.					
		2.1.6.	Thermal processing equipment (fryers, hot plate for tapiocas, pancakes, and others) exclusive for gluten-free food.					
	**2.2.**	**Furniture (tables, workbenches, window displays, shelves)**						
		2.2.1.	Furniture designed for easy cleaning (smooth, without wrinkles and cracks, and of a waterproof material).					
		2.2.2.	Existence of specific furniture for the production of gluten-free food or existence of a proper cleaning process between the use of the furniture for gluten-containing and gluten-free food proved by an updated registration worksheet with information consistent with what is being observed.					
	**2.3.**	**Kitchenware**						
		2.3.1.	Kitchenware of material, size, and shape that allow easy cleaning.					
		2.3.2.	General kitchenware (pans, spoons, knives, cutlery, etc.) exclusive for gluten-free food, stored in an appropriate and identified place, in organized manner, and protected against contamination by gluten or, when not exclusive, properly sanitized prior to the usage and preparation of gluten-free food.					
		2.3.3.	Difficult to clean kitchenware (sieves, pastry brush, graters, etc.) exclusive for the production of gluten-free food.					
	**2.4.**	**Cleaning of equipment, machinery, furniture, and kitchenware**						
		2.4.1.	Equipment, machinery, furniture, and kitchenware kept in proper hygienic-sanitary conditions, that is, without the presence or accumulation of residues, with proof by means of registration in specific spreadsheets, updated, and with information consistent with what is observed.					
		2.4.2.	Availability of cleaning products required to perform the operation and dilution, contact time, and form of use/application according to the instructions recommended by the manufacturer.					
		2.4.3.	Availability and suitability of all necessary utensils to carry out the cleaning operation with those in good condition.					
		2.4.4.	Whenever gluten-free food is handled, cleaning of equipment, machinery, furniture, and kitchenware that are of common use for gluten-free and gluten-containing foods is performed properly.					
		2.4.5.	Use of an exclusive sponge or similar to sanitize all kitchenware, equipment, and surfaces that will come into contact with gluten-free food.					
		2.4.6.	Dishwasher usage: crockery used for gluten-containing and gluten-free food sanitized at different moments.					
**3.**	**Food service employees**							
	**3.1.**	**Clothing**						
		3.1.1.	Employees display proper personal cleanliness: body cleanliness, clean hands, short nails, clean uniforms.					
		3.1.2.	Employees use a uniform exclusive for handling gluten-free food or a uniform which has not been previously used to handle food with gluten, without having been washed afterwards.					
	**3.2.**	**Hygienic habits**						
		3.2.1.	There is guidance (posters) for proper hand hygiene, which includes appropriate moments and procedures, accessible to employees and followed correctly.					
		3.2.2.	Employees do not handle gluten-containing and gluten-free foods simultaneously or engage in any act that could lead to cross-contamination, such as eating during food preparation.					
	**3.3.**	**Employees training program and supervision**						
		3.3.1.	Existence of a proper and continuous training program related to the production of gluten-free food and registration of these trainings.					
		3.3.2.	Existence of supervision of the procedures to avoid gluten contamination by a properly trained supervisor.					
**4.**	**Food production and transport**							
	**4.1.**	**Raw materials, ingredients, and package**						
		4.1.1.	Raw materials, ingredients, and packaging are inspected at the reception, observing if the labels of the raw material and ingredients meet the specific legislation for gluten. Potential sources of gluten are identified and controlled upon reception.					
		4.1.2.	Defrosting of gluten-free food held in a separate location from gluten-containing food and without getting in touch with utensils and equipment where gluten-containing food is stored or held in locations that are cleaned before procedure.					
	**4.2.**	**Selection of recipes and ingredients and food preparation**						
		4.2.1.	The selection of recipes and ingredients and the manufacturing technical cards are accurately followed for gluten-free food, with the label of all ingredients being checked at the time of preparation.					
		4.2.2.	Water or oil previously used in the preparation of gluten-containing food is not reused at the preparation of gluten-free food.					
		4.2.3.	Ingredients are not of common use for the production of gluten-free and gluten-containing food (e.g., margarine). All products intended for the preparation of gluten-free food are identified.					
	**4.3.**	**Production flow**						
		4.3.1.	The reception of gluten-free products occurs in a separated space from other products or carried out at a different moment.					
		4.3.2.	Segregation or separation of procedures such as production scheduling or specific/exclusive lines for gluten-free food, with an ordered flow without crossing between gluten-free and gluten-containing food.					
	**4.4.**	**Labeling and storage of final product and/or semi-prepared products**						
		4.4.1.	Final and/or semi-finished products (products that will be used in the elaboration of pasta, fillings, sauces, etc.), packaged in a suitable container (known composition of the container material—gluten-free), intact and exclusive for gluten-free food.					
		4.4.2.	Labeling statements with visible identification and in accordance with current legislation regarding the presence or absence of gluten.					
		4.4.3.	Products with and without gluten stored separately by a physical barrier or proper distance, in order to avoid contact between them.					
	**4.5.**	**Transportation of the final product**						
		4.5.1.	Transportation maintains the integrity of food.					
		4.5.2.	The vehicle does not simultaneously carry gluten-containing and gluten-free food or it does carry these products simultaneously, but with due care of separation by physical barrier or proper distance between them (use of sealed containers, of impermeable material).					
**5.**	**Distribution**							
	**5.1.**	At the distribution of food, employees follow procedures to eliminate the risk of gluten contamination, through hand hygiene, use of protective utensils, and disposable gloves and others whenever there is previous contact with gluten-containing food.						
	**5.2.**	Separate disposal, at different distribution counters. Preparation according to the presence/absence of gluten.						
	**5.3.**	Preparation identified with labels or other visible method according to its gluten content.						
	**5.4.**	Kitchenware used for serving food exclusive for gluten-free preparation and identified with different colors.						
	**5.5.**	Monitoring of the preparation identification plates in regards to the presence/absence of gluten at the moment of distribution.						
**6.**	**Documentation**							
	**6.1.**	**Manual of good practices**						
		6.1.1.	Operations carried out at the facility are in accordance with an on-site Good Practices Manual that meets the legal requirements in regards to content and updating.					
	**6.2.**	**Proper hygienization of furniture and facilities in order to prevent gluten contamination**						
		6.2.1.	Existence of Standard operating procedures established for this item, which are being fulfilled.					
	**6.3.**	**Proper hygienization of surfaces, equipment, and kitchenware in order to prevent gluten contamination**						
		6.3.1.	Existence of SOPs established for this item, which are being fulfilled.					
	**6.4.**	**Food recall program**						
		6.4.1.	Existence of SOPs established for this item, which is being fulfilled.					
**7.**	**Responsibility and authority**							
	**7.1.**	Responsibilities and authorities are defined and communicated within the organization to ensure effective operation and maintenance of the gluten contamination control.						
**8.**	**Coordinator of the food safety team**							
	**8.1.**	Top management has a Gluten Contamination Control Team Coordinator.						
	**8.2.**	The designated Coordinator has the responsibility and authority to administer the Gluten Contamination Control Team and to organize their work.						
	**8.3.**	The designated Coordinator has the responsibility and authority to ensure relevant training and education of all members of the gluten contamination control team.						
	**8.4.**	The designated Coordinator has the responsibility and authority to ensure that the gluten contamination control system is established, implemented, maintained, and updated.						
**9.**	**Methods for comunication in the gluten contamination control**							
	**9.1.**	The organization ensures that the team is informed in proper time of changes of raw materials, ingredients, and services.						
	**9.2.**	The organization ensures that the team is informed in proper time of changes in production systems and equipment.						
	**9.3.**	The organization ensures that the team is informed in proper time of changes in production facilities, location of equipment, and surroundings.						
	**9.4.**	The organization ensures that the team is informed in proper time of changes in cleaning and sanitation programs.						
	**9.5.**	The organization ensures that the team is informed in proper time of changes in levels of staff qualification and/or designation of responsibilities and authorities.						
	**9.6.**	The organization ensures that the team is informed in proper time of changes in knowledge regarding gluten contamination and control measures.						
	**9.9.**	The organization ensures that the team is informed as soon as possible in the event of a consumer complaint indicating a possible risk of gluten contamination in the food.						
	**9.10.**	The organization ensures that the team is informed in proper time of any circumstances or occurrences not covered in the previous items that may have an impact on the control of gluten contamination.						
	**9.11.**	The team ensures that any information relevant to the control of gluten contamination is always updated in the system by the responsible party and passed on to the rest of the employees.						
**10.**	**Flow charts**							
	**10.1.**	Flowcharts are prepared for categories of products or processes (implemented) by the gluten contamination control system.						
	**10.2.**	Flowcharts are clear, precise, and sufficiently detailed.						
	**10.3.**	Flowcharts are checked on site by the gluten contamination control team and verification records are kept.						
**11.**	**Treatment of potentially unsafe products**							
	**11.1.**	The organization treats non-compliant products preventing them from entering the food production chain or attesting the presence of gluten on the label of such foods in case of possible contamination.						
	**11.2.**	All food produced that may have been affected by a nonconformity situation is kept under the control of the organization until it has been evaluated.						
	**11.3.**	The organization notifies interested parties when products that are no longer under the organization’s control are subsequently determined to be unsafe (contaminated with gluten), initiating the recall process.						
